# Naturally-occurring canine invasive urothelial carcinoma harbors luminal and basal transcriptional subtypes found in human muscle invasive bladder cancer

**DOI:** 10.1371/journal.pgen.1007571

**Published:** 2018-08-08

**Authors:** Deepika Dhawan, Noah M. Hahn, José A. Ramos-Vara, Deborah W. Knapp

**Affiliations:** 1 Department of Veterinary Clinical Sciences, College of Veterinary Medicine, Purdue University, West Lafayette, IN, United States of America; 2 Departments of Oncology and Urology, Johns Hopkins University School of Medicine, Baltimore, MD, United States of America; 3 Johns Hopkins Greenberg Bladder Cancer Institute and the Sidney Kimmel Comprehensive Cancer Center, Johns Hopkins University School of Medicine, Baltimore, MD, United States of America; 4 Department of Comparative Pathobiology, College of Veterinary Medicine, Purdue University, West Lafayette, IN, United States of America; 5 Purdue University Center for Cancer Research, West Lafayette, IN, United States of America; University of Leeds, UNITED KINGDOM

## Abstract

There is growing evidence that molecular subtypes (e.g. luminal and basal subtypes) affect the prognosis and treatment response in patients with muscle invasive urinary bladder cancer (invasive urothelial carcinoma, iUC). Modeling these subtypes in pre-clinical animal studies is essential, but it is challenging to produce these subtypes, along with other critical host and tumor features, in experimentally-induced animal models. This study was conducted to determine if luminal and basal molecular subtypes are present in naturally-occurring canine iUC, a cancer that mimics the human condition in other key aspects. RNA sequencing was performed on 29 canine treatment naive iUC tissue samples and on four normal canine bladder mucosal samples. Data were aligned to CanFam 3.1, and differentially expressed genes were identified. Unsupervised hierarchical clustering of these genes revealed two distinct groups (n = 13, n = 16). When genes that distinguish basal and luminal subtypes in human cancer (n = 2015) were used to probe genes differentially expressed between normal canine bladder and iUC, 829 enriched signature genes were identified. Unsupervised hierarchical clustering of these genes revealed two distinct groups comprised of 18 luminal subtype tumors and 11 basal subtype tumors. The enriched genes included *MMP9*, *SERPINE2*, *CAV1*, *KRT14*, and *RASA3* in basal tumors, and *PPARG*, *LY6E*, *CTSE*, *CDK3*, *and TBX2* in luminal tumors. In supervised clustering, additional genes of importance in human iUC were identified in canine iUC associated with claudin-low and infiltrated tumors. A smaller panel of genes (n = 60) was identified that distinguished canine luminal and basal iUC with overall 93.1% accuracy. Immune signature patterns similar to those in human iUC were also identified with the greatest enrichment of immune genes being in the basal subtype tumors. These findings provide additional compelling evidence that naturally-occurring canine iUC is a highly relevant and much needed model of human iUC for translational research.

## Author summary

Approximately 50% of patients with invasive urinary bladder cancer (invasive urothelial carcinoma, “iUC”) die from their cancer. Better therapeutic strategies are essential. A relatively recent important finding in iUC is the presence of molecular subtypes (luminal and basal subtypes). These subtypes affect the cancer aggressiveness and response to particular treatments. To make progress against iUC, these subtypes must be modeled in animal studies which are used to select the most promising new treatments for human trials. It is, however, challenging to replicate these subtypes combined with other key features in experimentally-induced tumors. We performed RNA sequence analyses of naturally-occurring iUC in pet dogs (with comparison to normal canine bladder and sequencing data from humans), and demonstrated that the luminal and basal subtypes are clearly present in canine iUC. This builds on previous findings of the similarities between iUC in dogs and humans in pathology, metastatic behavior, and response to chemotherapies. In conclusion, the findings of our study provide further compelling evidence that canine iUC is a highly relevant model of human iUC for translational research. Canine clinical trials can be used to select the most promising strategies to take into human trials, thus benefiting humans as well as pet dogs.

## Introduction

Muscle invasive bladder cancer or more specifically, invasive urothelial carcinoma (iUC), is lethal in approximately 50% of patients, with most deaths being due to drug-resistant metastatic disease [1]. Clearly better therapies for iUC are needed. One of the key advances that could improve iUC therapy has been the identification of molecular subtypes (luminal, basal) which are linked to iUC behavior and response to chemotherapy, targeted agents, and immunotherapies [2–10]. Basal iUC is inherently aggressive, is associated with more advanced local stage and metastasis at diagnosis, and is enriched for *STAT3*, *TP63*, *KRT5/6A*, *CD44*, *HIF-1*, and *EGFR* [2–10]. Luminal iUC is thought to be associated with better clinical outcomes, and is enriched for *ER*, *TRIM24*, *FOXA1*, *GATA3*, *FGFR3*, and *PPARG* [2–10]. Subclassifications within these major subtypes have also been identified [9,10]. New drugs and drug combinations delivered in the context of molecular subtypes could greatly improve the outlook for patients with iUC.

Relevant pre-clinical animal models that accurately predict drug effects in humans are needed to optimize new treatment protocols for iUC [11,12]. This is especially the case now as the number of bladder cancer patients is insufficient to test even part of the potential new drug and combination therapy protocols of interest. Studies in relevant pre-clinical models could be used to screen multiple approaches with the most promising ones taken into human trials. These animal models must recapitulate the molecular subtypes of iUC found in humans, as well as mirroring other aspects of the human condition (heterogeneity, innate and acquired drug resistance, metastasis, etc.). In addition, with the resurgence of immunotherapies, and the recognition that the immune response plays an important role in the efficacy of other cancer therapies as well, it is critical that the animal model also have a level of immunocompetence similar to that in human cancer patients [13,14]. While it is difficult to create an experimental animal model that has these collective features, there is growing evidence that naturally-occurring iUC in pet dogs can serve as this essential relevant model to complement traditional experimental models of bladder cancer [11,12]. Invasive UC in dogs closely mimics the cancer in humans in pathology, molecular features, biological behavior including sites and frequency of distant metastasis, and response to chemotherapy [11,12]. Proteomic and genomic analyses have defined further intriguing similarities between iUC in dogs and humans [15,16]. Differentially expressed genes (between iUC and normal bladder) that are shared between dogs and humans include >450 genes identified by microarray, and >800 identified by RNA-seq (P<0.05; 2FC) [15,16]. Relevant to the importance of molecular subtypes, early microarray studies of canine iUC have indicated the presence of luminal and basal subtypes similar to those in human iUC [15]. Though vast similarities exist between the human and dog disease, there are also expected differences. Activating mutations in the MAPK pathway occur in iUC in both species, but BRAF mutations which predominate in dogs, are rare in humans [11].

Clinical trials in dogs with iUC are considered a win-win scenario with each dog having the chance to benefit, and new knowledge gained that will help humans and dogs. With canine iUC comprising approximately 2% of the estimated 6 million new canine cancer cases each year, there are ample numbers of dogs for translational research. In addition to treatment trials, canine iUC can be used to elucidate environmental exposures and gene-environment interactions involved in the cancer development. Exquisitely high breed-associated risk, such as the 20X increased risk in Scottish Terriers compared to mixed breed dogs, offers unparalleled opportunities to investigate the causes of iUC, and to perform early detection / early intervention trials [12]. Chemical exposures (lawn chemicals, older flea control products) have been associated with increased iUC risk in dogs, and consumption of vegetables in addition to commercial dog food has been associated with a lower risk of iUC in dogs [12].

RNA-seq analysis of canine iUC was performed to confirm and extend earlier findings. Specifically, the aims of the work were to: (1) determine the presence of the major luminal and basal transcriptional subtypes in canine iUC, (2) explore the presence of additional subclassifications within subtypes as reported in humans, (3) define a smaller set of genes which could be used to subtype canine iUC, and (4) investigate the immune signatures present in canine iUC. The study was successfully completed, and the findings provide further compelling evidence that naturally-occurring iUC in dogs can serve as a highly relevant model of human invasive bladder cancer.

## Results

### Unsupervised clustering of differentially expressed genes between canine normal urothelial cells and iUC segregate canine iUC into distinct transcriptional subtypes

**Fig 1:**
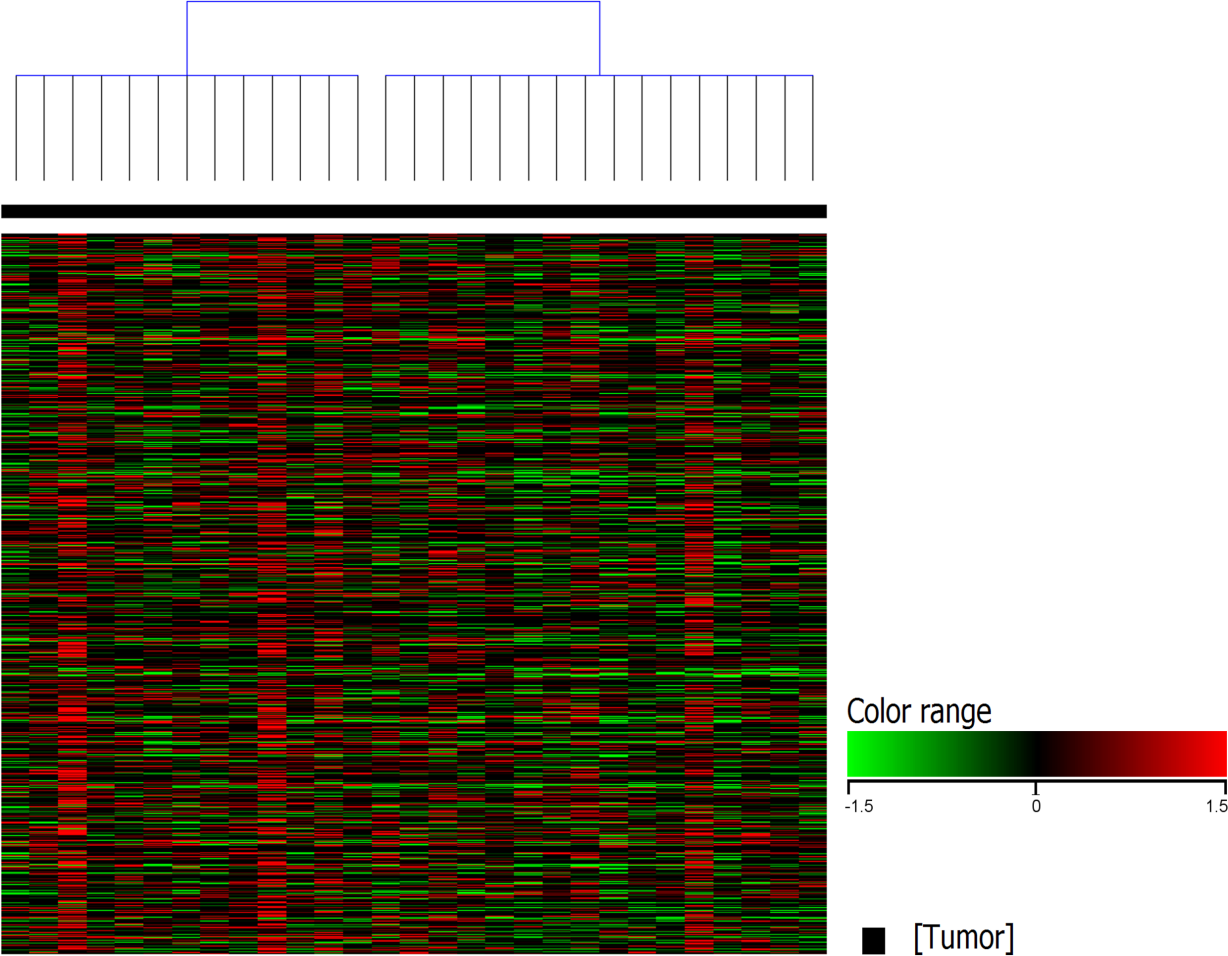
Unsupervised clustering of canine iUC samples separates tumor samples into two distinct groups. Canine iUC samples were normalized using TMM and DESeq concurrently. Statistical analyses were conducted using edge R (on TMM normalized data) and DESeq2 (on DESeq normalized data) with p corr ≤0.05. Only genes with ≥2 fold change were selected from each analysis, and the two lists of differentially expressed genes were combined. These differentially expressed genes were hierarchically clustered using K-means to result in unsupervised clustering of samples. The analyses revealed two distinct groups within the canine iUC tumor dataset (n = 13, n = 16).

**Fig 2:**
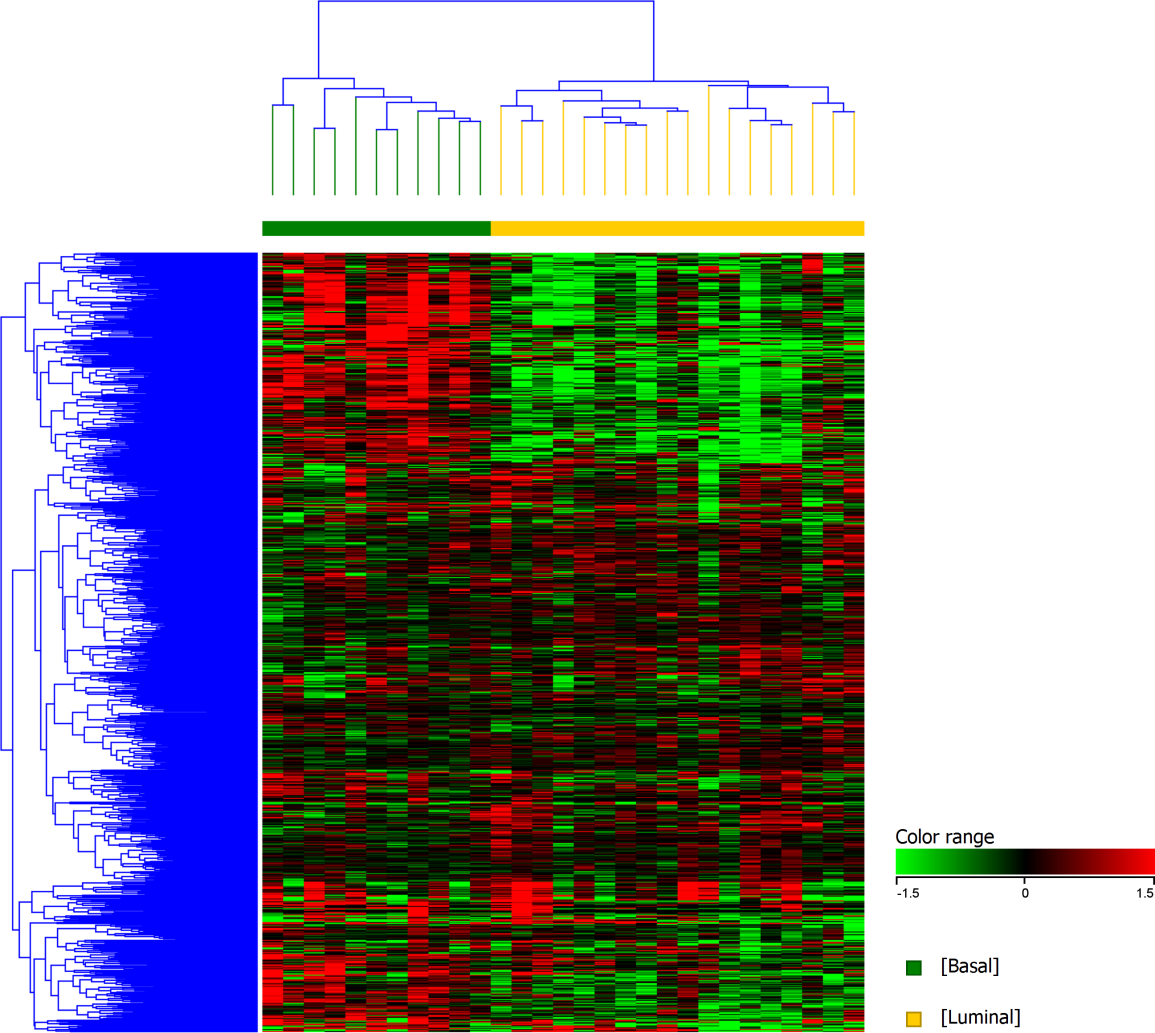
Unsupervised hierarchical clustering of genes identifying basal and luminal subtypes in canine iUC samples. Genes that assign basal and luminal subtypes in human breast cancer (and confirmed in human iUC) [6] were used to query the differentially expressed genes identified in canine iUC samples, and 829 commonly expressed genes were identified. Unsupervised hierarchical clustering was performed using these 829 genes employing Euclidean distance metrics and Ward’s linkage algorithm as a distance metric. Two distinct groups were identified as basal (n = 11) and luminal (n = 18).

Unsupervised clustering of canine iUC samples segregated canine iUC into two distinct groups (n = 13 and n = 16) (Fig 1). Hierarchical clustering of genes that are differentially expressed in basal and luminal subtypes in human breast cancer (n = 2015; of which 829 were present in the canine dataset), and confirmed to be of relevance in human iUC [9], revealed two distinct groups within the canine tumor samples (Fig 2). The group with the larger number of tumors (n = 18, 62% of tumors) was enriched for genes reported in human luminal iUC with examples including *PPARG*, *FOXA1*, *CTSE*, *CDK6*, *TBX2*, and *SNCG* [1,4,5,8]. The second group (n = 11, 38% of tumors) was enriched for genes reported in human basal iUC including *MMP9*, *SERPINE2*, *RASA3*, *and PLAUR* [5,6,8]. It is pertinent to state that the tumors identified as basal (n = 11), were the same tumors as present in the smaller group using K-means clustering (n = 13). Differentially expressed genes commonly identified in the canine and human iUC samples in The Cancer Genome Atlas (TCGA) included, but were not limited to, *PIGR*, *AGR2*, *CDK3*, *ACTG2*, *DES*, *TPM2*, *TAGLN*, and *MYH11* [4,17].

### Enrichment of genes in canine iUC that assign luminal, basal, and infiltrated phenotype in human iUC

**Fig 3:**
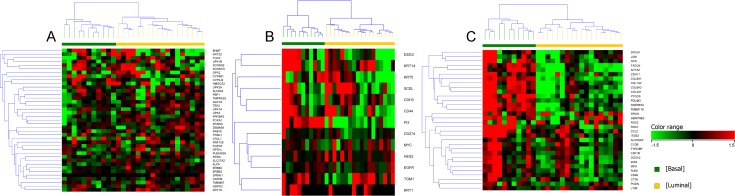
Supervised clustering of genes implicated in human iUC that assign luminal, basal and infiltrated phenotype to tumors. A list of reference genes reported to assign luminal (A), basal (B), and infiltrated (C) subtypes in human iUC was used to query the gene expression patterns in the canine iUC dataset. Supervised clustering of the data displays similar patterns of these genes in canine iUC as reported in human iUC [5,8,17].

Supervised clustering of genes reported to be of importance in subclassifying subtypes in human iUC confirmed the presence of the gene signatures in canine iUC, and included genes in addition to those enriched in the pairwise comparison. Genes of particular interest because of their relevance to human luminal iUC included *PPARG*, *VGLL1*, *and LY6E* [5,8]. The expression of these genes was heterogenous across canine luminal iUC samples (Fig 3A). Similar heterogeneity was observed when examining *PI3*, *DSG3*, and *KRT14* in canine basal iUC samples (Fig 3B) [5,8]. Most of the genes reported to be associated with assigning “infiltrated” human iUC were found to be upregulated in the canine basal iUC samples [8], although *SRGN*, *SERPINE2*, and *RGS2* were enriched in some of the canine luminal iUC samples (Fig 3C).

### Enrichment of genes in canine basal iUC that assign claudin-low subtype in human iUC

**Fig 4:**
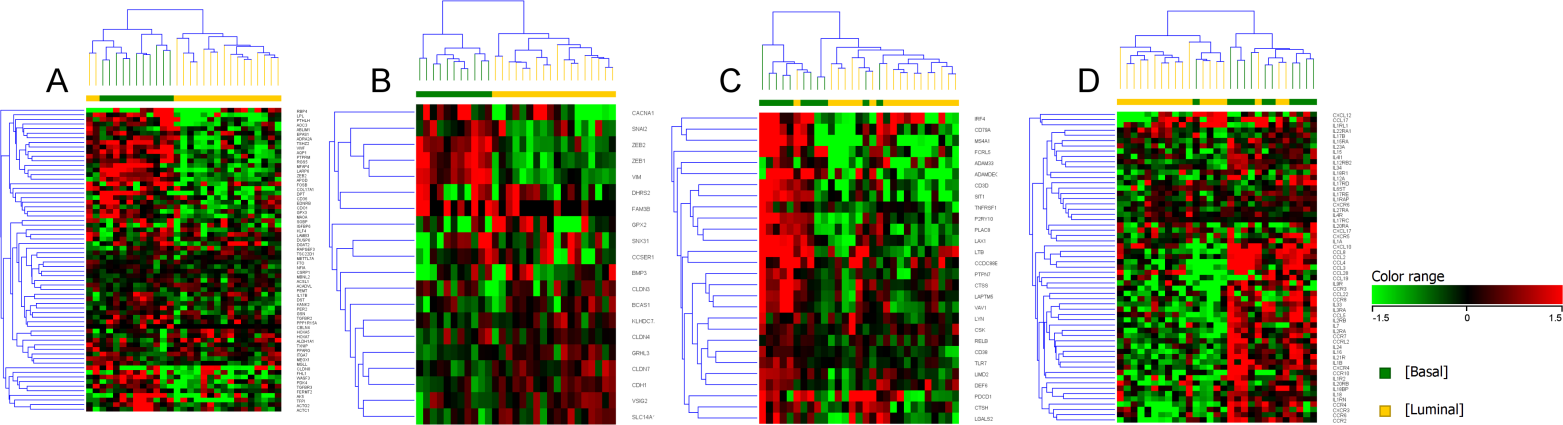
Supervised clustering of genes implicated in human iUC that assign claudin-low features in tumors. A list of reference genes reported to assign claudin-low subtype in human breast cancer (A), human iUC (B), enriched immune signatures associated with claudin-low subtypes (C), and enriched chemokines and cytokines (D) in human disease was used to query the gene expression patterns in the canine iUC dataset [18,19]. Supervised clustering of the data displays features resembling claudin-low patterns in the majority of canine basal iUC as reported in human iUC.

Hierarchical clustering of bladder tumors using a list of genes that was previously utilized to define claudin-low tumors in human breast cancer patients, confirmed basal tumors to be enriched for claudin-low genes [18]. Two luminal tumors also exhibited enrichment of genes associated with claudin-low subtype (Fig 4A). In the panel of genes relevant to the claudin-low transcriptional subtype (Fig 4B), *CLDN7*, *CLDN3*, and *CDH1* were upregulated in canine luminal iUC, and *SNAI2*, *ZEB1*, and *ZEB2* were upregulated in canine basal iUC [8,17,18]. The enriched immune-suppressive signatures associated with claudin-low (in human breast cancer) and the enriched chemokines and cytokines enriched in human iUC (Fig 4D) further confirmed the claudin-low subtype in the majority of canine basal (and one luminal) tumors [18,19].

### Enrichment of genes in the *p53*, *p63*, and *PPARG* pathways in canine iUC

**Fig 5:**
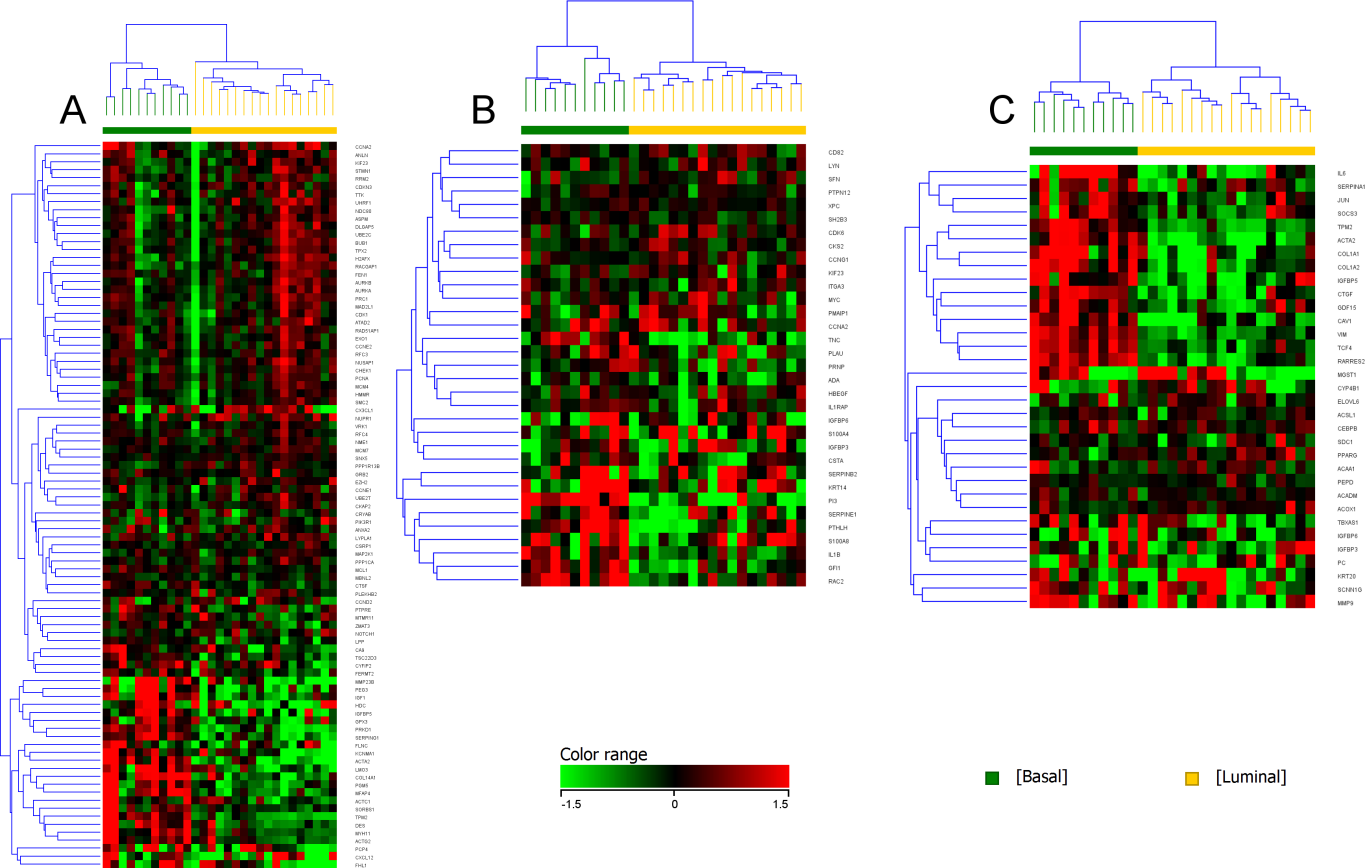
Supervised clustering of genes implicated in pathways of importance in human iUC. Genes reported to be of importance in *p53* (A), *p63* (B) and *PPARG* (C) pathways in human iUC were assessed in the canine iUC transcriptional subtypes. Normalized intensity values were used for supervised hierarchical clustering using Euclidean distance metrics and Ward’s linkage algorithm as a distance metric. Supervised clustering of the data reveals similar patterns of these genes as reported in human iUC.

The *p53* signature genes [3,5] were enriched in some of the luminal and basal tumors, as reported in human iUC (Fig 5A). For example, *IGF-1*, *SERPING-1*, *MFAP4*, *LMO3*, and *PGM5* were enriched in canine basal tumors, whereas *CCNE2*, *CX3CL1*, *FEN1*, and *UHRF1* were found to be enriched in canine luminal tumors. *P63* pathway genes such as *TNC*, *PI3*, *SERPINE-1*, *S100A8*, *GFI1*, and *RAC2*, were enriched in canine basal tumors (Fig 5B) [3,5]. Some of the genes upregulated in the *PPARG* pathway in basal tumors in human iUC, i.e., *COL1A1*, *COL1A2*, *CAV1*, and *ACTA2*, were also upregulated in canine basal tumors (Fig 5C) [5].

### Predictive transcriptional subtype panel of genes for segregating canine luminal and basal iUC tumors

**Fig 6:**
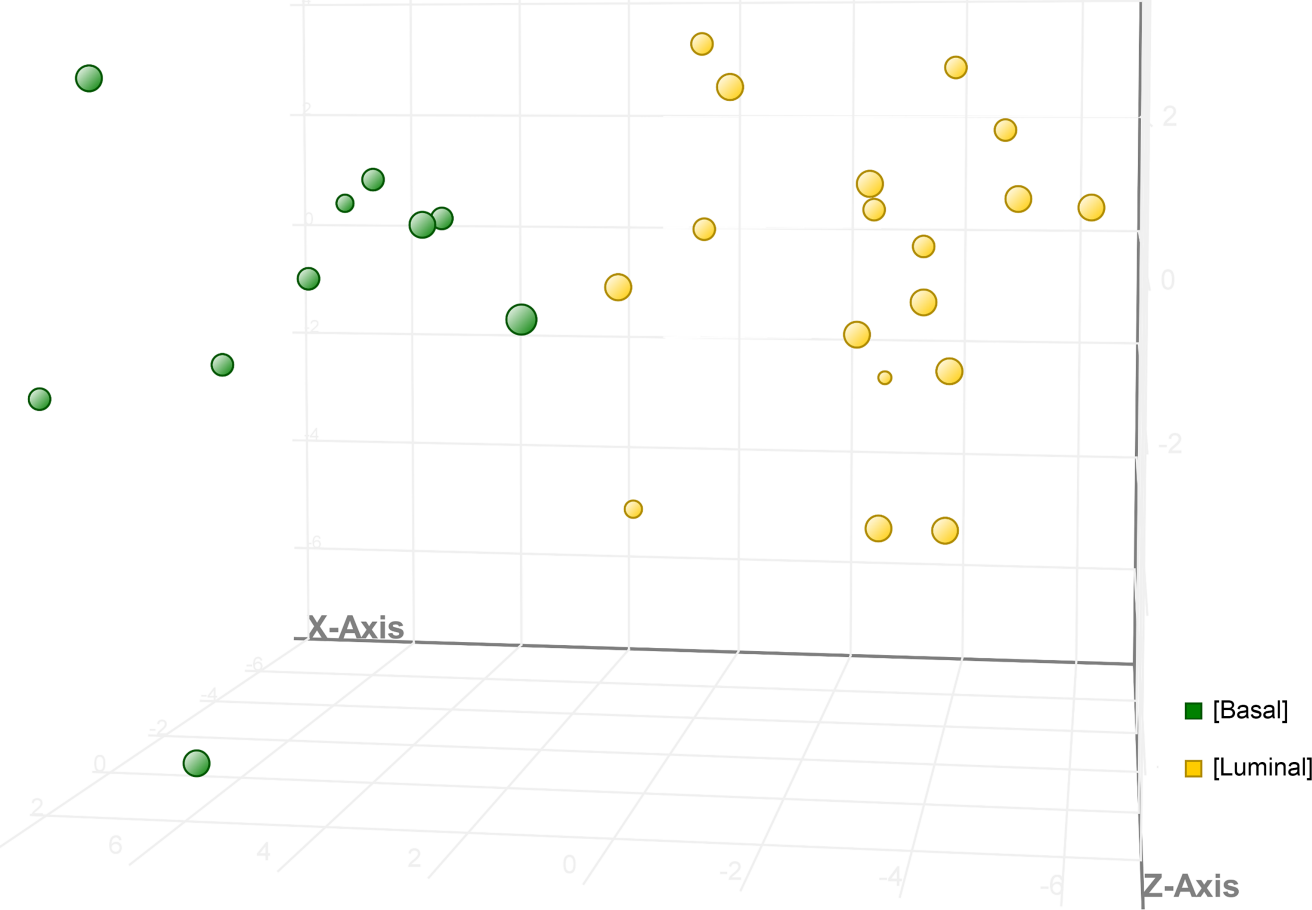
Class prediction model using a panel of genes selected to segregate canine iUC into basal and luminal subtypes. A smaller panel of genes of importance in characterizing basal or luminal subtypes in human iUC was used to develop a class prediction model using Support Vector Machine (SVM) alogrithm and using validation parameters with 100,000 iterations and using validation type “Leave One Out” to generate the validation algorithm outputs. This allowed the selection of a group of genes (n = 60) which could be used to assign canine iUC samples to luminal or basal subtypes. The PCA plot depicts the segregation of tumors as identified by SVM. Of particular interest are genes also implicated in human iUC such as *PPARG*, *CTSF*, *LY6E*, *VGLL1*, *SERPINE2*, *SULT1A1*, and *CAV1*.

A panel of genes (n = 60) was developed as a class prediction model that could segregate canine iUC into basal or luminal subtypes (Fig 6A). The validation algorithm output predicted tumors with basal and luminal transcriptional subtype with an overall accuracy of 93.1%. Basal tumors were predicted with an accuracy of 100% (n = 11/11), and luminal tumors were predicted with 88.9% accuracy (n = 16/18). Of particular interest are genes also implicated in human iUC including *PPARG*, *CTSF*, *LY6E*, *VGLL1*, *SERPINE2*, *SULT1A1*, *and CAV1* (Fig 6). The PCA plot shows clear segregation of basal and luminal subtypes (Fig 6) [3,5,6,8,10,17].

### Enrichment of immune signatures in the majority of canine basal iUC samples

**Fig 7:**
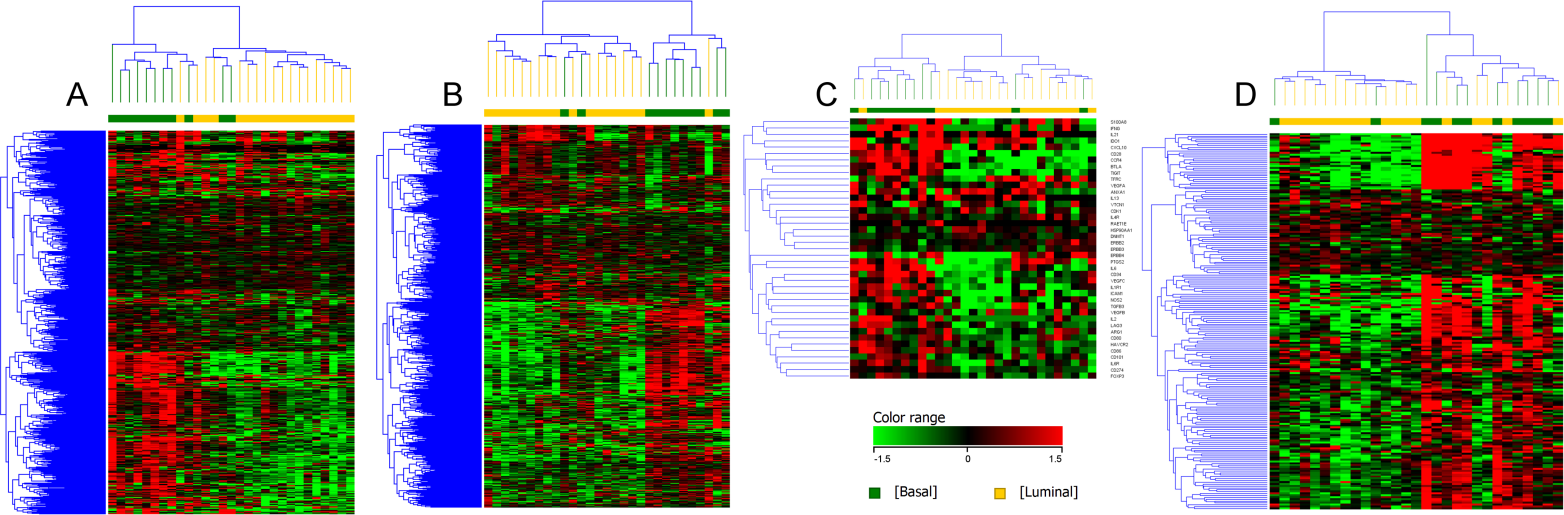
Expression patterns of immune signatures in canine basal and luminal iUC. A list of immune signature genes from different immune cell types, checkpoint inhibitors, antigens and genes covering both adaptive and innate immune response (nCounter, Nanostring, PanCancer Immune Profiling Panel, Seattle, WA) (A), interferon-γ inducible genes (from the Interferome database [22] (B), immunosuppressive genes regulating the function of myeloid derived suppressor cells (MDSCs) and regulatory T-cells (C), and genes enriched in immune system process identified from GO:0002376 (D) were used to visualize the gene expression patterns that exist in canine iUC. Normalized intensity values were used for supervised hierarchical clustering using Euclidean distance metrics and Ward’s linkage algorithm as a distance metric. Note the higher expression of immune genes in the canine basal iUC samples (A,B,C,D). Two basal tumors were found to be immune “cold”, and a minority of luminal tumors were identified as immune “hot”.

When analyzing a list of genes (n = 595) associated with immune profiling of human cancers in the canine iUC samples, immune signatures were enriched in 9 of 11 basal tumors as compared to luminal tumors (Fig 7A) [20]. Interferon-γ (IFN-γ) inducible genes (n = 694) were also found to be enriched in canine basal tumors (Fig 7B) [21]. Genes that are involved in immunosuppressive functions, i.e., those regulating the function of myeloid derived suppressor cells (MDSCs) and regulatory T-cells, and genes identified using GO:0002376 (immune system processes) were also found to be enriched in basal tumors. It was noted that two tumors identified as having the basal subtype did exhibit immune cold signatures. Variable number of luminal tumors were found to be enriched for immune signatures (1/18 as visualized in 7A, 7B and 7C, and 4/18 as depicted in 7D).

## Discussion

The most important outcome of the study was the finding of distinct luminal and basal subtypes in canine iUC. It is becoming widely accepted that these subtypes have critical bearing on the clinical behavior of iUC and response to therapy in humans, and thus must be modeled in pre-clinical animal models [10,19,22]. Luminal iUC commonly has *FGFR3*, *ERBB2*, and *ERBB3* activating mutations, and is thought to generally be associated with a better prognosis [10,23]. Basal iUC is enriched with *EGFR* and *HIF-1* expression, is often metastatic at presentation, and can possess squamous and sarcomatoid histological features and epithelial-to-mesenchymal transition cell biomarkers [10,23]. In canine iUC, 62% of tumors were luminal, and 38% were basal. These percentages are similar to those reported in human iUC [10].

It is noted that different reports in the literature have classified human iUC into two to six subtypes including some subclassifications of the two major (luminal, basal) subtypes [2,5,6,10,17,24]. Multiple studies have focused on TCGA data [4,10,17]. In a 2014 report of TCGA data from the first 131 samples, tumors were classified into four subtypes, referred to as clusters I-IV, with clusters I and II being luminal, and clusters III and IV being basal [4]. In the 2017 report of TCGA data from the larger set of 412 samples, iUCs were categorized into five subtypes including luminal papillary (similar to previous cluster I), luminal infiltrated (similar to previous cluster II), luminal, basal/squamous (similar to previous clusters III and IV), and neuronal subtypes [10]. Within the basal subtype, earlier reports also identified a subset referred to as claudin-low tumors characterized by *RB* mutations, *EGFR* amplification, and low expression of *FGFR3* and *PPARG* [6,17,19]. These tumors had enhanced immune signatures, but more immunosuppressive features (e.g. immune checkpoints) than immune enhancing features. Although our study of canine iUC did not reveal subtypes beyond luminal and basal subtypes, the finding of claudin-low and infiltrated features in some of the canine tumors suggest that these subtypes could exist in canine bladder cancer and may be elucidated with larger numbers of cases in follow-up studies. Regardless of the numbers of subtypes in human iUC, there is consensus that the most critical distinction is between luminal and basal subtypes, as occurs in canine iUC [10,22,24].

The characterization of subtypes is important because the prognosis and treatment response are thought to be impacted by the subtype of the cancer [4,10,19,22]. It is anticipated that future therapies could be tailored to the individual patient based in part, on the subtype of their cancer. It is recognized that basal tumors are inherently more aggressive and are associated with shorter overall survival than luminal tumors, yet basal tumors appear more sensitive to cisplatin-based therapies than luminal tumors. [3,4,5]. Additional subtypes also could affect treatment outcome. Luminal papillary tumors (previously included in TCGA cluster I) which have *FGFR3* mutations, fusions and amplifications, but an underactive immune environment may be best treated with *FGFR3* inhibitors [10]. Luminal infiltrated tumors (previously included in TCGA cluster II) would be expected to respond to immune checkpoint inhibitors, but less so to cisplatin-based regimens [10]. There is evidence for this in that patients with locally advanced and metastatic iUC refractory to platinum chemotherapy were more likely to respond to the anti-PD-L1 immune checkpoint inhibitor, atezolizumab, if they had luminal cluster II tumors [25]. Similarly, in a study in which iUC were assigned to subtypes using the “UNC” classification scheme, luminal tumors had the best overall survival with or without neoadjuvant chemotherapy [8]. Patients with basal tumors appeared to gain the most improvement in overall survival from neoadjuvant cisplatin-based chemotherapy [8].

In human iUC, claudin-low tumors have been reported to have poor overall survival regardless of treatment [8]. Claudin-low tumors are characterized by a stromal phenotype, enrichment of epithelial-to-mesenchymal transition (EMT) markers, immune response genes, and lack of luminal differentiation markers [18,19]. It has been proposed that claudin-low tumors express high levels of cytokines and chemokines normally repressed by PPARG [19]. The enrichment of immune signatures, in particular PD-CD1 in claudin-low tumors, reinforces the need to stratify these patients to potentially receive immune checkpoint inhibitor treatment as opposed to basal tumors that could respond better to chemotherapy [19].

Canine basal tumors were found to be enriched for *p63* pathway genes as reported in human basal transcriptional subtype [3]. Both canine luminal and basal iUC tumors expressed patterns associated with *p53* target genes, as previously reported in humans [3,5]. Although the *p53* phenotype was originally suggested to be a third subtype, this is now being reconsidered [3]. It is recognized that the contribution of stromal cells could have been driving the p53 signature in some of the tumors in earlier analyses [3].

An intriguing finding in the canine iUC samples, was the presence of different RNA-seq immune signatures between the luminal and basal subtypes. Identifying transcriptional immune signatures in tumors offers a tool to complement other methods (microscopy, immunohistochemistry, flow cytometry, and others) in analyzing the immune response to cancer and to cancer therapy [22]. There is renewed and growing recognition that the immune response impacts patient outcome, response to immunotherapies, and response to other types of treatments [26]. In melanoma and non-small cell lung cancer, for example, the tumor immune state is recognized for prognostic value and for predictive value in the response to immune checkpoint blockade therapies [27,28]. Immunotherapies are rapidly shifting the treatment priorities for bladder cancer, as well as other human cancers [29]. Within the last two years, there has been a dramatic shift in the treatment of metastatic iUC with the approval of five therapies targeting the programmed cell death protein (PD-1)/programmed cell death ligand 1 (PD-L1) axis [30]. As described in other cancers types, it is expected that the pre-existing tumor immune landscape in iUC will impact the response to chemotherapy as well as to immunotherapies. Molecular subtyping of the cancer could allow more precise prognostication, and the selection of therapies most likely to help each individual patient [31]. With the dramatic effects of immune checkpoint inhibitors only occurring in approximately 20% of patients, tools to assist in predicting and monitoring outcomes and in selecting patients most likely to benefit from immunotherapy are critical [30]. In human iUC, immune transcriptomic analyses have revealed different immune signatures of prognostic relevance in the molecular subtypes [22]. These immune signatures were also found in canine iUC.

RNA-seq data from the canine iUC samples were interrogated using four panels of immune genes. The nCounter PanCancer Immune Profiling Panel and GO analyses (GO:0002376 immune system processes) provide a broad picture of the immune landscape by incorporating several hundred genes from different immune cell types, common checkpoint inhibitors, tumor antigens, and other genes covering both the innate and adaptive immune response [20]. The third panel of genes used to interrogate the immune landscape in canine iUC consisted of 694 IFN-γ inducible genes from the Interferome database [21]. Interferons have a central role in anti-tumor immune responses, and are emerging as prognostic and predictive biomarkers of chemotherapy, as well as immunotherapy [32]. In analyses of human iUC samples, some of the most enriched biological processes included responses to IFN-γ, especially in cluster IV tumors [21]. In the canine samples, notable enrichment in IFN-γ inducible genes was also observed, especially in basal tumors. The fourth panel used consisted of immunosuppressive genes regulating the function of myeloid derived suppressor cells and regulatory T-cells (compiled from GSEA database). Immunosuppressive genes were also found enriched in basal tumors, further supporting the use of immunotherapy in basal iUC. Future work will focus on specific immune features in the canine tumors.

Another key finding from the study was the identification of a smaller subset of genes (60 genes) that can be used to categorize canine iUC as luminal or basal. This is similar to the finding of a small subset of 47 genes which could be used to distinguish human luminal and basal subtypes [6]. As larger canine iUC data sets become available, this panel of 60 genes can be further validated, and can expedite further studies.

The findings in this study further increase the value of the naturally-occurring canine model of iUC. Dogs with naturally-occurring iUC have previously been appreciated as a model for human iUC because of similarities in histopathology, molecular features, biological behavior (local invasion, distant metastases in 50% of cases), prognostic factors, and response to chemotherapy [11,12]. Clinical trials in pet dogs are quite feasible as most dog owners appreciate the access to new therapies for their pet, the opportunity to help generate new knowledge that will benefit other dogs and humans, and the financial assistance that often accompanies canine clinical trials [12]. New therapies and new combinations of therapies can often be evaluated in a frontline setting because there is not a highly effective, well defined or regimented standard of care that must be used in dogs [11,12]. In addition, most dog owners are open to allowing cystoscopy before and during treatment to obtain tissue samples for biological endpoints, and many dog owners will allow an autopsy and tissue collection if the dog is euthanized due to declining quality of life from the cancer or comorbid conditions. The work reported here adds to the value of the canine model by demonstrating the presence of luminal and basal subtypes, and suggesting a different immune landscape between the two subtypes. Furthermore, the identification of a canine 60-gene expression signature capable of distinguishing luminal versus basal tumor subtypes provides the opportunity, with further validation, to design a feasible point-of-care cross-species assay suitable for simultaneous parallel comparative oncology investigations in both canine and human bladder cancer patients to further optimize outcomes in both species.

In conclusion, this study provides compelling evidence for the presence of luminal and basal subtypes in naturally-occurring canine iUC, a finding that further increases the value and utility of this disease as a highly relevant model for translational research to improve the outcome of humans with iUC. Future work in larger iUC datasets is warranted to investigate additional subtypes, validate the 60-gene panel for distinguishing luminal and basal iUC, correlate subtypes to clinical outcome, and apply the canine iUC model in high impact translational research.

## Materials and methods

### Ethics statement

With consent from the owners of dogs with invasive urothelial carcinoma who were undergoing cystoscopy as part of their diagnostic evaluation, a small tissue sample collected during cystoscopy was saved for RNA sequencing analysis. This was approved by the Purdue Animal Care and Use Committee (Approval Number 1111000169).

### Experimental methods

#### Canine tissue samples

All tissues from dogs were collected with the approval of the Purdue Animal Care and Use Committee. Canine iUC tissues (n = 29) were collected by cystoscopic biopsy from treatment-naive dogs (n = 29), and samples were stored in trizol at -80°C. The tissues were selected sequentially as dogs presented for cystoscopic biopsies, and dogs of any age, gender or breed were included. Each tumor was obtained from a unique dog, i.e. one tumor sample sequenced per dog. Only tissues with histopathologic confirmation of iUC were processed for RNA isolation and included in the study. All tumors included in the study were high grade tumors [12] and had >95% epithelial cellularity. Since the majority of tumor samples were cystoscopic biopsies obtained with instruments small enough for use in dogs, the size of the samples was limited. Therefore, it was not consistently possible to assess the depth of invasion. None of the tumors exhibited squamous or adenoid differentiation [33]. Normal canine bladder tissue samples (n = 4) were collected from dogs that were being euthanized for conditions other than bladder related diseases. The urothelial layer was stripped from full thickness bladder using fine dissection and stored immediately in trizol (Invitrogen, Carlsbad, CA) at -80°C for subsequent RNA isolation.

#### Whole transcriptomic sequencing of normal and canine iUC tumor samples using RNA sequencing

Total RNA was isolated following manufacturer’s instructions (Invitrogen, Carlsbad, CA) and purified using RNAeasy (Qiagen, Valencia, CA). All samples were processed for RNA-seq and run in one batch at the Biomedical Genomics Core at Nationwide Children’s Hospital, Columbus, Ohio. The quality of total RNA was evaluated using NanoDrop spectrophotometer (NanoDrop Technologies, Wilmington, DE) and Agilent Bioanalyzer (Agilent Technologies, Santa Clara, CA). RNA quality was considered acceptable if the RNA integrity number was seven or greater. Ribosomal-RNA was removed from total RNA with Ribo-Zero rRNA removal kit (Illumina), and mRNA libraries constructed (ScriptSeq v2 RNA-Seq library preparation kit, Epicentre Biotech, Madison, WI). Following purification, di-tagged cDNA was amplified by limit-cycle PCR and purified using AMPure XP System (Beckman Coulter) [34,35]. Paired-end 150 bp sequence reads were generated using Illumina HiSeq 4000 platform, to obtain an average of 50 million reads/sample. Raw reads were cleaned for PCR artifacts and adapter trimmed, then aligned to the canine genome (CanFam 3.1 reference genome) using COBWeb to obtain expression levels for annotated genes and isoforms (Strand NGS, v3.1, Build 235027, Agilent Technologies, Santa Clara, CA) [36].

#### Statistical analyses

Data were processed, and statistical analyses conducted using Strand NGS [36]. Raw RNA-Seq data were subjected to normalization (separately using TMM and DESeq), filtered by read metrics, and subjected to quantification. Pairwise comparison was performed using edge R (on TMM normalized data) and DESeq2 (on DESeq normalized data) with Benjamini-Hochberg FDR multiple testing correction (p < 0.05; 2-fold or higher change) comparing normal mucosa/urothelial layer samples vs canine iUC samples. Differentially expressed genes identified using both methodologies were pooled for subsequent analyses. Unsupervised clustering was performed on the tumor samples using K-means. Transcriptional subtypes were assigned by hierarchically clustering the normalized canine intensity values against genes with known expression patterns in human breast cancer [6,37]. Subclassification of transcriptional subtypes, i.e., luminal, basal, claudin-low, infiltrated was performed by hierarchically clustering the canine iUC database using genes of known importance in human iUC [3,5,8]. The canine iUC dataset was hierarchically clustered using genes enriched in *p53*, *p63*, *PPARG* pathways and genes of importance in human iUC [3,5]. Immune signatures were examined in canine iUC samples by hierarchically clustering genes of known importance in cancer (nCounter PanCancer Immune Profiling Panel) [20] and IFN-γ inducible genes from the Interferome database [21]. Immune genes enriched in basal and luminal tumors were identified by the GO term GO:0002376. Hierarchical clustering was performed for visualization of the differentially expressed genes using Euclidean distance metrics and Ward’s linkage algorithm as a distance metric. A panel of genes featuring the most significant luminal and basal genes to classify transcriptional subtypes in canine iUC samples was developed using Support Vector Machine alogrithm and using validation parameters with 100,000 iterations and using validation type “Leave One Out” to generate the validation algorithm outputs. Principal component analyses (PCA) plot was used to visualize the luminal and basal tumors.

## Supporting information

S1 Table List of genes (n = 829) used to identify basal and luminal subtypes in canine iUC samples. (XLSX)

S2 Table List of genes used to assign luminal, basal and infiltrated tumors. (XLSX)

S3 Table List of genes used to assign claudin-low subtype in canine iUC. (XLSX)

S4 Table List of genes used to associate p53, p63 and PPARG pathways in canine iUC. (XLSX)

S5 Table List of genes selected to segregate canine iUC into basal and luminal subtypes. (XLSX)

S6 Table List of genes used to elucidate immune signatures in canine basal and luminal iUC. (XLSX)
